# Single-institution experience with Shape medical polymer sponge embolization as adjunct therapy for rapid aortic remodeling in the multi-modal management of complex persistent large false lumens following aortic dissection

**DOI:** 10.1016/j.jvscit.2025.101913

**Published:** 2025-07-07

**Authors:** Richa Kalsi, Anahita Shiva, Charlyn Gomez, Aidan P. Wiley, Georges Jreij, Mirnal Chaudhary, Shahab Toursavadkohi

**Affiliations:** a Division of Vascular Surgery, Department of Surgery, University of Maryland Medical Center, Baltimore, MD; b Department of Surgery, University of Maryland School of Medicine, Baltimore, MD

**Keywords:** Chronic dissections, False lumen, Shape memory embolization plug

## Abstract

Persistent false lumen (FL) pressurization after endovascular repair of aortic dissection is associated with increased aortic morbidity and mortality. Shape memory polymer sponge devices (IMPEDE-FX, Shape Medical) have shown promise in promoting sac regression after endovascular aortic repair in abdominal aortic aneurysms in ongoing trials. Here, we report the adjunct use of these devices as part of a multimodal approach to achieve FL occlusion in three patients (average age, 59 years; male = 2, female = 1) with limited options for the management of their persistent large FL following type A (n = 1) and type B (n = 2) aortic dissections between March and December 2024. FL thrombosis and aortic remodeling were achieved in all three patients based on postoperative imaging within 1 to 6 months after intervention. There was one mortality in a Marfan’s patient secondary to Zone 0 aortic rupture 2 months postoperatively, distant from areas of previous intervention. Our early clinical experience with the Shape memory sponge in managing large, complex persistent FL is encouraging, but further prospective studies are needed.

False lumen (FL) patency after standard endovascular repair (TEVAR) of type B aortic dissection (TBAD) is associated with aortic expansion in 30% of patients, increased reintervention rates, and decreased long-term survival.^1^, ^2^, ^3^ Current management options aim to either prevent distal retrograde flow into the FL, such as endo-trashing or the Knickerbocker technique, or promote a thrombogenic environment in the FL through embolization with coils, plugs, or glue. Novel devices have recently become available that promote the rapid formation of organized thrombus with collagen deposition to facilitate the obliteration of a flow lumen and promote favorable remodeling. In this case series, we report the off-label use of a Shape memory polymer device (IMPEDE-FX Embolization Plug/Shape Memory Plug [SMP]), which is United States Food and Drug Administration-approved for use in the peripheral vasculature and is currently being investigated in ongoing trials in the promotion of sac regression after endovascular repair of abdominal aortic aneurysms (EVAR).^4^ This polymer was used as an adjunct to achieve FL occlusion and rapid remodeling of large recalcitrant FLs in three patients with a history of multiple previous endovascular and open interventions for complex post-dissection aneurysms. Patients consented to inclusion in our case series.

## Case 1

A 79-year-old man with a distant history of a Zone 3 to 10 TBAD originally managed with Zone 3 to 4 replacement with tube graft via left thoracotomy in 2013 at an outside hospital was transferred to our institution after imaging demonstrated degeneration of the aorta in multiple locations, notably a 6.8-cm thoracic aneurysm and a bi-lobed 8-cm infrarenal aortic aneurysm. Please see Supplementary Table I (online only) for detailed surgical history and device specifications. The aorta had remodeled such that the right iliac system was fed through the FL, the left iliac system was fed through the true lumen (TL), and the visceral segment was perfused through multiple fenestrations in a thickened chronic dissection septum. This was managed in a two-stage procedure to decrease the risk of spinal cord ischemia (SCI). Infrarenal aortic stenting was performed in a double-barrel fashion in the TL and FL to exclude the infrarenal aneurysm, followed by Zone 3 to 5 TEVAR 5 months later. Notably, on postoperative day (POD) 7 from the TEVAR, the patient experienced a delayed presentation of proximal lower extremity weakness and numbness concerning for SCI, which resolved with conservative management. Four months later, the patient’s aorta had increased to 7 cm with ongoing endoleak, so the decision was made to intervene. The patient first underwent candy plugging of the FL, but the sac continued to expand, so Zone 2 to 5 TEVAR with thoracic branch endoprosthesis and additional candy plug placement in the FL was performed 7 months later. After all these interventions, the FL remained pressurized with a persistent type IB endoleak, and the descending thoracic aorta reached a size of 11.1 cm. At this point, a final attempt at sac embolization with SMP was offered vs no further intervention. The patient elected to proceed with one more embolization attempt.

**Fig 1:**
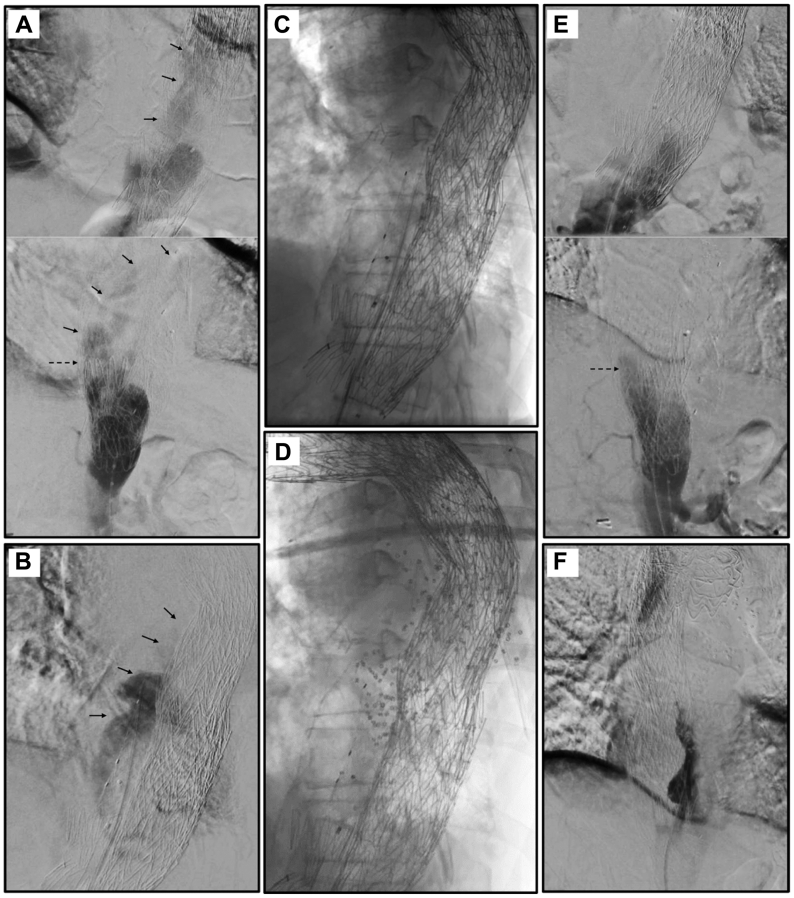
Case 1 operative images. **(A)** Aortograms from two obliquities demonstrating type Ib endoleak with contrast at the paravisceral segment passing briskly into the false lumen (FL) past the top of the candy plugs into the thoracic aortic sac (*small black arrows* demonstrating contrast blush into the FL, *dashed arrow* demonstrating top of candy plug in the FL for reference). **(B)** Angiogram from within the FL with *small black arrows* demonstrating large blush extending up towards the thoracic aorta. **(C)** Long working sheath positioned between the two candy plugs in the FL. **(D)** The FL after deployment of 200 IMPEDE-FX plugs with visible radiopaque markers. **(E)** Completion aortograms from the same starting obliquities demonstrating no further contrast extending upwards into the FL. *Dashed arrow* again shows the top of the candy plug for reference. **(F)** Angiogram from within the FL demonstrating contrast stasis at the tip of the catheter instead of free flow into the FL, as seen in **(B)**.

**Fig 2:**
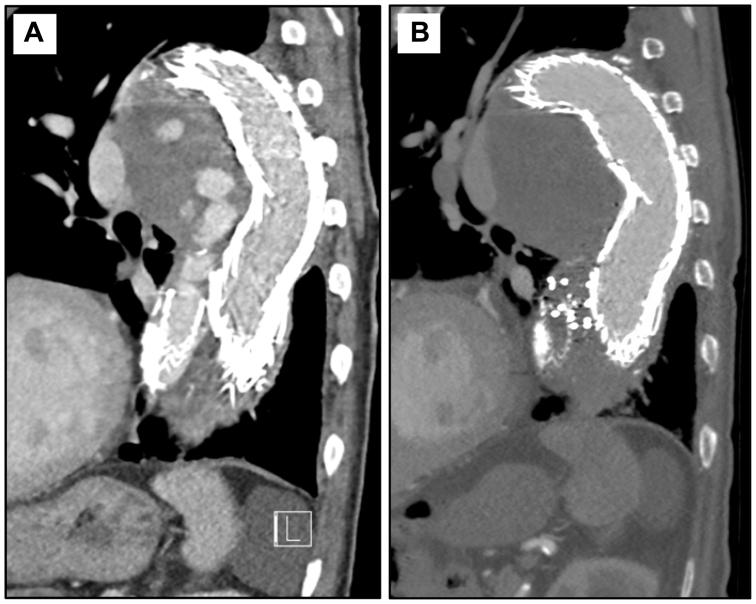
Case 1 computed tomography angiograms. **(A)** Preoperative sagittal view of the false lumen (FL) with extensive endoleak noted. **(B)** Six-month postoperative sagittal view at the same level, demonstrating resolution of the endoleak and thrombosis of sac.

Under general anesthesia, the bilateral groins were accessed, and multiple aortograms were obtained demonstrating contrast at the paravisceral segment flowing past the candy plugs in the FL (Fig 1, *A*). The FL was accessed using a working wire and angled catheter, and the wire was advanced between the two candy plugs into the residual sac and confirmed with intravascular ultrasound (IVUS) and angiogram (Fig 1, *B*). A long 6 Fr working sheath was advanced into the FL (Fig 1, *C*), and 200 SMPs were deployed using the Shape Memory IMPEDE-FX RAPIDFILL device, which can deploy five IMPEDE-FX embolization plugs at a time. (Fig 1, *D*). Completion aortograms demonstrated significantly decreased filling of the FL (Fig 1, *E*), and FL angiograms demonstrated contrast hanging near the tip of the catheter (Fig 1, *F*). The patient was discharged on POD 2. One-month interval computed tomography angiography demonstrated resolution of the endoleak, and 6-month interval computed tomography angiography demonstrated a decrease in the size of the sac to 10.4 cm from 11.1 cm (Fig 2, *A* and *B*).

## Case 2

A 65-year-old woman with a history of Marfan’s disease had extensive aortic history, which began with a TBAD from a Kommerell diverticulum (proximal Zone 3) to Zone 10 in 2012, complicated by severe visceral malperfusion requiring open retrograde right common iliac artery to superior mesenteric artery bypass. Please see Supplementary Table II (online only) for detailed surgical history and device specifications. Three years later (2015), the patient developed degeneration of Zone 2 to 3 with an increase in descending aortic size to 6.5 cm from ∼4 cm in 2012. This was managed in conjunction with cardiac surgery, with debranching with bilateral common carotid artery to subclavian artery (SCA) bypasses followed by ascending aorta to bilateral common carotid artery bypasses as well as Zone 1 to 5 TEVAR. The origins of the left SCA and aberrant right SCA were plugged with Amplatzer plugs. The patient also had an enlarging complex aortobi-iliac aneurysm, which, in 2016, was repaired with a combination of Endologix and thoracic GORE grafts and left hypogastric coiling. The patient remained stable under combined cardiac and vascular follow-up for the subsequent 8 years until she was noted to have aneurysmal degeneration of the uncovered paravisceral aorta reaching 5.8 cm and right common iliac artery aneurysm of 4.5 cm in 2023. For SCI protection, the patient underwent a thoracic aorta to T10 intercostal artery bypass ahead of endovascular extent II repair with three-vessel fenestrated EVAR and right iliac branch endoprosthesis. Unfortunately, the patient developed a persistent type 2 endoleak in the mid-thoracic aorta after the 2023 repair, with overall diameter increasing to 6.5 cm from 5.8 cm in 2024. An endovascular extent II repair had already been completed, and open options were very high risk given the patient’s extensive abdominal and thoracic surgical history, so a plan was made to embolize the sac with multiple modalities, including SMP.

**Fig 3:**
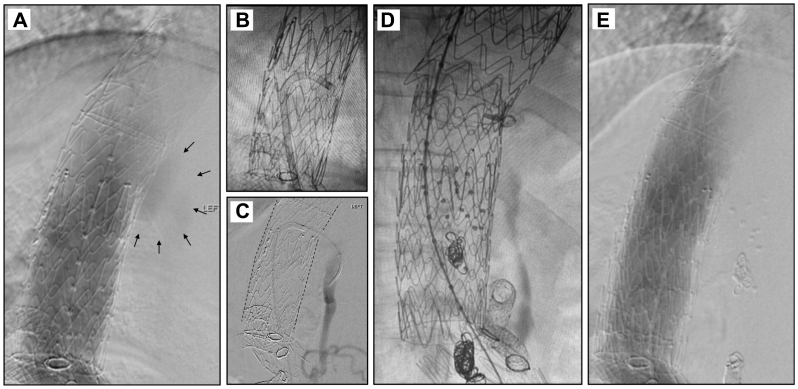
Case 2 operative images. **(A)** Initial aortogram demonstrating endoleak into the false lumen (FL) as demonstrated by *small black arrows*. **(B)** Steerable sheath in the true thoracic aortic lumen within endograft directed posteriorly towards the FL. **(C)** Access of the T11 intercostal artery via laser fenestration (*dotted lines* demonstrating endograft, *gap* indicating the fenestration). **(D)** Status post coiling of both feeding intercostal arteries and deployment of IMPEDE-FX plugs. **(E)** Completion aortogram demonstrating no continued flow into the FL.

**Fig 4:**
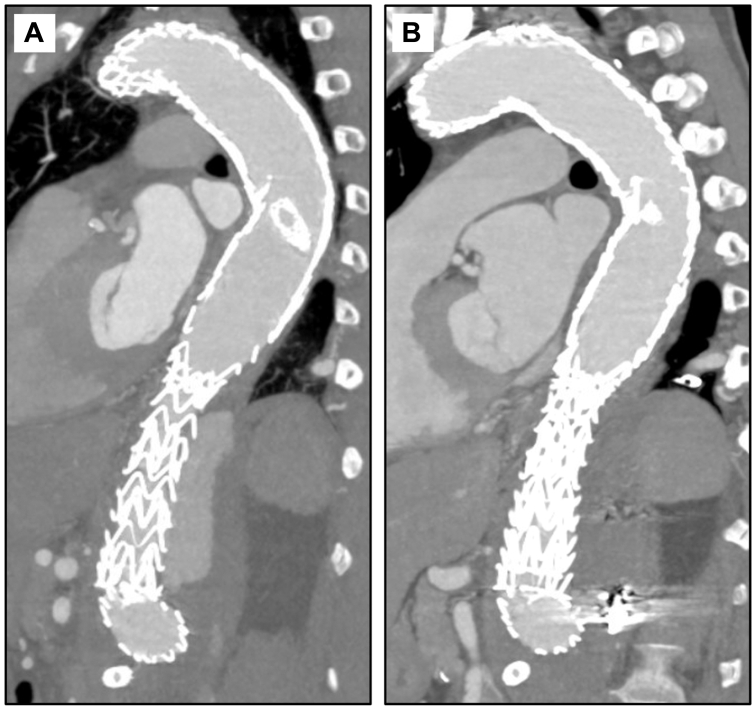
Case 2 computed tomography angiograms. **(A)** Preoperative sagittal view shows extensive endoleak into the false lumen (FL). **(B)** One-month postoperative sagittal view showing interval resolution of endoleak with some sac remodeling around Shape memory plugs (SMPs) and candy plug within the thrombosed FL.

Under general anesthesia with neuromonitoring, a 22 Fr DrySeal was placed over a stiff wire into the thoracic aorta via right groin access. Thoracic aortography and IVUS demonstrated a type II endoleak feeding the FL (Fig 3, *A*). The FL was accessed with laser fenestration and a 7 Fr steerable sheath (Fig 3, *B*). Angiography through the FL demonstrated the orifices of the feeding T11 intercostal arteries, which were embolized with Terumo Hydrocoils (Fig 3, *C* and *D*). The FL cavity was then filled with 10 SMPs (Fig 3, *D*), and the fenestration was covered with an endovascular cuff. Completion angiogram demonstrated resolution of the endoleak with no further FL filling (Fig 3, *E*). The patient was discharged on POD 1, and 1-month postoperative imaging demonstrated complete resolution of the endoleak with FL thrombosis and overall diameter reduction to 6 cm from 6.5 cm (Fig 4, *A* and *B*). Unfortunately, 2 months later, the patient suffered a Zone 0 rupture of the proximal untreated ascending aorta and subsequently passed away.

## Case 3

The third patient was a 33-year-old man with hypertension who had an acute type A dissection with right lower extremity malperfusion and underwent open total arch replacement with the Thoraflex system as well as right external iliac artery and proximal common femoral artery stenting. Please see Supplementary Table III (online only) for detailed surgical history and device information. This patient presented 1 month later with abdominal complaints with paravisceral collapse of the TL with visceral malperfusion managed with Zone 3 to 5 TEVAR. One-month postoperative imaging did not demonstrate significant endoleak. However, 2 months later, the patient presented again with a heart failure exacerbation, at which time cross-sectional imaging demonstrated extensive endoleak and growth of the aorta by 3 mm over 1 month. The patient had a left pleural effusion, which was tapped, and the output was serosanguinous. With concern for possible impending rupture and to prevent further rapid sac expansion, the patient was urgently taken to the operating room with a plan to thrombose the FL with a combination of endo-trash and polymer plugs.

**Fig 5:**
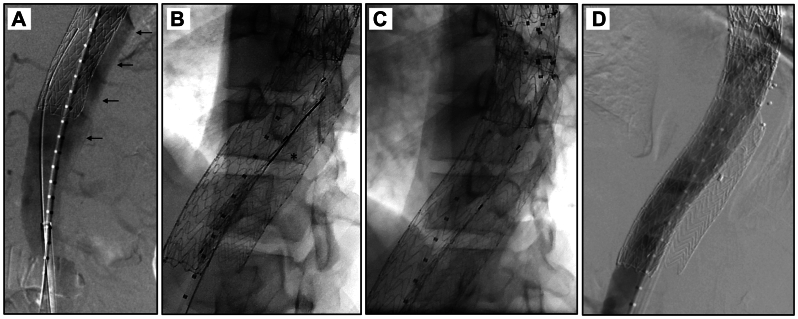
Case 3 operative images. **(A)** Starting aortogram demonstrating aggressive flow into the thoracic aortic false lumen (FL). *Small black arrows* point to extensive contrast opacification outside the edges of the endograft. **(B)** Image after deployment of the iliac limb into the FL with wire access through the limb into the proximal thoracic FL. **(C)** Status post deployment of IMPEDE-FX plugs into the proximal thoracic FL. **(D)** Completion aortogram with an Amplatzer plug within the iliac limb and with no further contrast filling of the FL.

**Fig 6:**
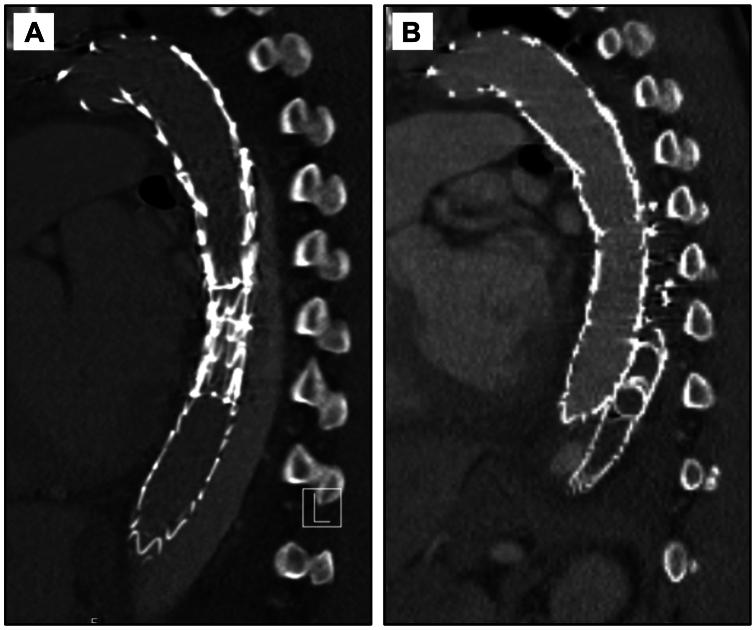
Case 3 computed tomography angiograms. **(A)** Preoperative image shows extensive endoleak into the false lumen (FL). **(B)** Three-month postoperative image showing interval resolution of endoleak with thrombosed FL.

Under sedation, both common femoral arteries were accessed. Angiography from the TL demonstrated aggressive bleeding into the FL extending into the thoracic aorta (Fig 5, *A*). After IVUS confirmation of position within the FL, a 16 Fr DrySeal was advanced over a stiff wire to deploy a Gore iliac extender in the FL at the level of the TEVAR (Fig 5, *B*). The inside of the Gore limb was accessed with a long working sheath to deploy 30 SMPs, followed by placement of an Amplatzer plug at the end of the Gore limb to prevent embolization of the polymer plugs (Fig 5, *C*). On angiography and IVUS, this completely occluded retrograde flow into the FL (Fig 5, *D*). Follow-up computed tomography scan at 3 months demonstrated no further flow into the FL, with extensive favorable remodeling (Fig 6, *A* and *B*).

## Discussion

This case series presents our early experience with the use of SMPs in the management of recalcitrant FL in three complex patients without many other options. The SMP is a porous biocompatible polymeric scaffold that expands from a crimped state into its final enlarged shape immediately upon exposure to an aqueous environment and the patient’s basal body temperature.^5^, ^6^, ^7^ Each plug contains a radiopaque marker and expands to 1.25 mL in final volume, depending on the device being used.^5^, ^6^, ^7^ Our institution used the IMPEDE-FX device, which was compatible with a 6 Fr sheath. The interconnected channels within the expanded plug are hydrophilic and support the formation of small, interconnected clots within the structure, rapidly generating an organized thrombus.^5^^,^^7^

The appeal of the SMP is that large animal studies suggest that the immune response to the SMP is distinct and more prone to favorable sac remodeling. Porcine models have demonstrated that, rather than an acute or chronic inflammatory response, a “healing phenotype” immune response is triggered by the SMP.^8^ This is defined by the increased presence of multinucleated giant cells surrounding the polymer, promoting fibrin and polymer degradation, collagen deposition, and mature connective tissue or scar formation.^7^^,^^8^ This contrasts with the immune response to platinum alloy bare metal coils, which is one of chronic inflammation surrounding a thrombus, and which may take longer to organize and form a scar.^7^ This is the basis for the recent United States Food and Drug Administration investigational device exemption approval of the application of an SMP device for pre-emptive abdominal aortic sac management to promote post-EVAR sac regression. An early single-arm study from the Netherlands demonstrated that, at 1 year after sac treatment with the SMP device, concurrent with EVAR, 81.8% of 34 patients achieved greater than 10% reduction in normalized aneurysm volume with no major adverse events.^9^

Patient 1 had undergone repeat interventions to cover entry tears proximally with a transient episode of SCI, which made increasing aortic coverage of an already anatomically complex aorta a poor option. Endo-trash attempts of the FL had not shut down the FL in the past, and open repair was not an option, given the patient’s frailty. This severely limited options for further management of the patient’s large 11.1-cm descending thoracic aorta to further FL-directed treatment. FL embolization with coils, nitinol or iliac occluder plugs, and physician-modified FL endografts serving as large FL occluders placed within the thoracic FL in a variety of configurations have been employed, particularly to manage retrograde flow from the abdominal aorta pressurizing thoracic FLs.^10^, ^11^, ^12^, ^13^ Technical success rates with aortic size reduction in 31% and aneurysm stabilization in 51% have been reported in the literature.^10^, ^11^, ^12^, ^13^ However, this leaves a large portion of patients—like patient 1—either failing traditional techniques or requiring reinterventions. With a large, pressurized space requiring embolization, our team felt that the SMP, which expands on contact with the blood and has a large surface area to promote rapid thrombus formation, would be advantageous. In particular, the hope was to achieve scar formation within the large FL to help stabilize the aorta and keep the FL sealed over time. For patient 1, extensive embolization of the FL with SMPs led to FL thrombosis at 6 months.

Patient 2 had already undergone extent II repair and had a hostile abdomen as well as a previous sternotomy and thoracotomy, with a type 2 endoleak from intercostal back-bleeding driving FL pressurization and expansion of in the descending thoracic aorta. The patient was closely followed by vascular and cardiac surgery, and FL-directed therapy was felt to be the best option. Here, standard coils were used in the relatively small space within the actual intercostal vessels to shut down flow. Given the patient’s connective tissue disease, adjunctive therapy to promote favorable sac remodeling was pursued with the use of the SMP, again due to the potential scaffolding effect for future scar formation within the sac. It is difficult to say whether similar results on follow-up imaging would be seen with coiling alone or SMP alone. Unfortunately, the patient suffered an unrelated Zone 0 rupture 2 months after her last operation, despite close follow-up in the aortic center. This was surprising, given that the maximum Zone 0 aortic diameter was 3.2 cm in 2012 and 4 cm in 2024. Zones 0 to 2 were uninvolved in the dissection and were used as the landing zone for the patient’s endovascular repairs. This underscores the clinical challenges of managing patients with Marfan’s disease.

Patient 3 had a pressurized, expanding FL with a blood-tinged effusion and was managed in a more urgent manner than patients 1 and 2. He was young but had recent open and endovascular aortic repairs, and had an ejection fraction of 15% going into the procedure, mandating an expedient strategy that would relatively rapidly decrease FL filling and promote favorable remodeling. Here, a traditional endo-trash technique was combined with SMPs. The iliac limb both occupied space in the FL and gave us the ability to place multiple SMPs though the limb, further away from aortic blood flow deep in the thoracic FL, followed by Amplatzer plug placement to prevent embolization of SMP back into the paravisceral aortic segment. The leak was shut down in the operating room, with favorable remodeling seen in the 3-month postoperative scan.

Given that 200 plugs were used in patient 1, understanding how many SMPs to use and the cost of each plug becomes very pertinent. The IMPEDE-FX is a single plug, and the IMPEDE-FX RAPIDFILL allows for the deployment of five plugs at a time. When a large volume needs to be filled and the risk of embolization is low, the IMPEDE-FX RAPIDFILL can be used to increase efficiency. The company provides volumetric analysis of the anticipated number of SMPs needed based on preoperative computed tomography imaging, which was largely accurate in our clinical experience in terms of the effect seen on intraoperative angiography. It is difficult to extrapolate the existing clinical experience in embolizing an excluded aneurysm sac after EVAR to a pressurized FL, as the physics of the two spaces are different. It stands to reason that FL embolization likely requires more plugs, given the active flow into the space. This highlights the importance of assessing each case individually and taking the volumetric analysis into account. In terms of cost, Shape Memory Medical provides a sliding scale pricing structure that caps the price as more devices are used. At our hospital, each plug was priced at approximately $1000 per plug, but after $10,000 (or 10 plugs), the charges were capped. This means that the cost per plug in cases 1, 2, and 3, respectively, was $50, $330, and $1000. In speaking with device representatives at our hospital for other standard coils, the manufacturers have pricing agreements in place to offset costs on bulk orders of coils or associated products, but the quoted cost of a single standard coil is $700 to $1000.

In our review of the literature, only one previous report described the application of the SMP to a patient with dissection. In that case, the SMP was used to embolize the FL of an infrarenal post-type A aortic dissection aneurysm with a maximum diameter of 81 mm.^5^ At 15 months in that study, the FL was fully thrombosed with remodeling of the RL.^5^ Because the three cases reported in the present study represent our experience in the use of SMPs in managing recalcitrant FLs, it is too early to draw broad conclusions regarding patient selection or to recommend SMP as a first-line therapy for certain FLs over others. The SMP served as a useful adjunct to help seal large pressurized FLs in complex patients who had either failed other interventions or had limited treatment options. Our early experience is promising, but further clinical experience and prospective studies are required.

## Conclusions

Our series of three complex cases show promise in the management of pressurized FL from chronic aortic dissections with a novel SMP device. However, further clinical investigations and shared experiences are required for broader generalizability.

## Funding

None.

## Disclosures

S.T. reports consulting for Artivion, Gore Medical, and Cook Medical.

## Appendix (online only)

Supplementary Table I Detailed surgical history for the patient described in Case 1.

Supplementary Table II Detailed surgical history for the patient described in Case 2.

Supplementary Table III Detailed surgical history for the patient described in Case 3.

Supplementary Video This video provides the intraoperative angiograms from which the still images in the figures were taken.
