# Supplementary material for: Single-institution experience with Shape medical polymer sponge embolization as adjunct therapy for rapid aortic remodeling in the multi-modal management of complex persistent large false lumens following aortic dissection
Source: J Vasc Surg Cases Innov Tech. 2025 Jul 7;11(5):101913. doi: 10.1016/j.jvscit.2025.101913 (PMC12361994; doi:10.1016/j.jvscit.2025.101913)
Supplement: Supplementary Table I — Detailed surgical history for the patient described in Case 1. [file mmc1.docx]

**Supplementary Table 1.**

| **CASE 1 Detailed Surgical History** | |
| --- | --- |
| **Month/Year** | **Indication/Procedure/Devices** |
| March 2013 | Zone 3-10 TBAD with aneurysm: Left thoracotomy, 30mmx34mm Gelweave tube graft replacement of Zone 3-4 (limited records available). |
| August 2022 | Stage I repair of Extent II Thoracoabdominal Aneurysm with dissection. Management of complex bilobed infrarenal aneurysm measuring 8cm. GORE cTAG 21mmx10cm into infrarenal aorta on right (FL), GORE bell bottom iliac extender 23mmx10cm into R CIA (FL); on left, GORE iliac extender 14mmx14.5cm into infrarenal aorta (TL) then GORE iliac extender 14.5mmx10mm into L CIA (TL) |
| January 2023 | Stage II repair of Extent II Thoracoabdominal Aneurysm with dissection. Management of 6.8cm descending thoracic aorta. TEVAR Zone 3-5, GORE cTAG 45mmx20cm, 45mmx20cm and 34mmx10cm. |
| April 2023 | Expansion of descending thoracic aorta to 7cm from 6.8cm previously, from retrograde flow from fenestrations. Cook TX2 device 32mmx70cm back table modified into candy plug shape, re-sheathed and deployed into distal thoracic aortic FL. |
| November 2023 | Expansion of descending thoracic aortic aneurysm suspected to be due to anastomotic degeneration of the proximal anastomosis of original open repair causing Type 1a endoleak of prior Zone 3-5 TEVAR, and Type 1b endoleak due to incomplete plug of FL from previous candy plug attempt. Zone 2 TBE (GORE TBE 45mmx15cm w/ 17mmx60mm branches x 2 for LSA), GORE cTAG 45mmx20cm x 2 to reline down to distal Zone 5. Another candy plug made with GORE Excluder limb 23mmx10cm and 22mm Amplatzer plug placed in FL next to previous candy plug to help seal the gutter. |
| March 2024 | Ongoing expansion of FL with descending thoracic aortic size now 11.1cm. IMPEDE-FX Embolization Plugs x 200 total placed into the FL using the Shape Memory IMPEDE-FX RAPIDFILL device. |
| Abbreviations: TBAD-Type B A FL- False Lumen. TL-True Lumen. CIA-Common Iliac Artery. LSA-Left Subclavian Artery. TEVAR-Thoracic Endovascular Aortic Repair. GORE-W.L. Gore & Associates, Flagstaff AZ. cTAG- Conformable Thoracic Stent Graft. TBE- Thoracic Branched Endoprosthesis. Cook- Cook Medical Inc. Shape Memory-Shape Memory Medical Inc., San Jose CA. | |
