# Supplementary material for: Single-institution experience with Shape medical polymer sponge embolization as adjunct therapy for rapid aortic remodeling in the multi-modal management of complex persistent large false lumens following aortic dissection
Source: J Vasc Surg Cases Innov Tech. 2025 Jul 7;11(5):101913. doi: 10.1016/j.jvscit.2025.101913 (PMC12361994; doi:10.1016/j.jvscit.2025.101913)
Supplement: Supplementary Table II — Detailed surgical history for the patient described in Case 2. [file mmc2.docx]

**Supplementary Table 2.**

| **CASE 2 Detailed Surgical History** | |
| --- | --- |
| **Month/Year** | **Indication/Procedure/Devices** |
| May 2012 | Zone 3-10 TBAD c/b severe visceral malperfusion requiring 170cm SBR, managed with open R CIA – SMA bypass, prolonged open abdomen |
| May 2015 | Degeneration of Zone 2-3, increase in size of descending thoracic aortic aneurysm to 6.5cm from ~4cm in 2012. Managed in conjunction with cardiac surgery with bilateral CCA to SCA bypasses(Gore Propaten 6mm ringed PTFE) followed by ascending aorta to bilateral CCA bypasses (10mmx8mmx8mm Terumo Vascutek trifurcated antegrade hybrid graft) via median sternotomy. Zone 1-5 TEVAR (GORE cTAG, 34mmx20cm, 34mmx10cm, and 34mmx15cm). One week later, patient was brought back to the OR for planned plugging of the origins of the L SCA and aberrant R SCA with Amplatzer plugs (1x10mm plug for LSCA, 1x12mm and 1x16mm plug for RSCA) via bilateral brachial access. Procedure staged due to excessive bleeding in first operation. |
| November 2015 | Type 2 endoleak identified around the Amplatz plugs in R SCA coming retrograde from the R CCA-SCA bypass filling the thoracic aortic sac. Given the patient’s connective tissue disease, decision was made to treat this with coil embolization of the origin of right replaced SCA via right radial access. |
| April 2016 | Complex aortobiiliac aneurysm noted to grow in diameter by 5 mm in 6 months to 4.5 cm. Given patient’s connective tissue history, decision made to intervene. Given extensive prior abdominal surgical history, endovascular repair performed. Coil embolization of left hypogastric artery, GORE iliac limb 12mmx14cm placed from aortic bifurcation to R hypogastric artery takeoff. Two 12mmx14cm GORE iliac limbs from aortic bifurcation to L EIA. Cook 28mmx30cm Endologix AFX main body device placed, then GORE cTAG 40mmx10cm x 2 built upward to below renal arteries. Additional 12cm GORE limb placed on L iliac AFX/GORE limb overlap area to prevent kinking. |
| September 2020 | Stenosis noted at the distal anastomosis of the R CCA-SCA bypass, balloon angioplasty performed |
| February 2021 | Ongoing stenosis noted at the distal anastomosis of the R CCA-SCA bypass, Boston Scientific 8mmx27mm bare metal stenting of the distal anastomosis |
| May 2023 | Patient developed degeneration of the paravisceral aorta with maximum diameter 5.8cm and a R CIA aneurysm of 4.5cm. The patient’s CCA-SCA bypasses had also both occluded at this point, placing the patient at exceedingly high risk for SCI with further aortic coverage. Therefore, a thoracic aorta to T10 intercostal artery bypass for SCI protection with GSV was performed via left thoracotomy followed by endovascular extent II repair with 3 vessel FEVAR with proximal TEVAR extension and R IBE. Left iliac extension was done with two 13mmx5cm Viabahn stents followed by 14.5mmx7cm stent. Right EIA was extended with a 14.5mmx12cm iliac limb. Infrarenal aorta was repaired with GORE Excluder 32mmx14.5cm, deployed from the left groin. The GORE Excluder junction with IBE was done with a 23 x 10 cm bell-bottom iliac extension. The ipsilateral limb was extended with Gore iliac extender 14.5mmx10 cm and 14.5mmx7cm iliac extenders. TEVAR was extended proximally to overlap with previous TEVAR using 34mmx100mm cTAG. The FEVAR was a 36mmx28mmx199mm Cook TX2 device with two 9mm fenestrations for the celiac and SMA and an 8mm fenestration for the R renal artery (L renal was previously occluded). R renal artery was stented with 7mmx29mm and 7mmx39mm VBXs, SMA and celiac each received 10mmx29mm and 10mmx39mm VBX stents. Two stents (8mmx38mm iCAST and 9mmx38mm iCAST) were placed to establish inflow to the intercostal bypass. |
| August 2024 | Patient’s mid thoracic aorta expanded further to 6.5cm with new Type 2 endoleak suspected to be secondary to backbleeding from intercostal arteries. Patient taken to OR for embolization of FL. Laser fenestraton used to access the FL, angiogram from FL showed T10 and T11 intercostal arteries filling, which were coil embolized using Terumo Hydrocoils. The FL was then filled with 10 Shape Memory IMPEDE-FX Embolization Plugs. Laser fenestration was covered with a cTAG 40mmx150mm. |
| Abbreviations: TBAD-Type B Aortic Dissection. FL- False Lumen. CIA-Common Iliac Artery. EIA-External iliac Artery. SMA-Superior Mesenteric Artery. CCA-Common Carotid Artery. SCA-Subclavian Artery. PTFE- polytetrafluoroethylene. TEVAR-Thoracic Endovascular Aortic Repair. cTAG-Conformable Thoracic Stent Graft. FEVAR-Fenestrated Endovascular Aortic Repair. GORE-W.L. Gore & Associates, Flagstaff AZ. Cook- Cook Medical Inc. Shape Memory-Shape Memory Medical Inc., San Jose CA. Terumo-Terumo Medical Corporation. | |
