# Supplementary material for: Single-institution experience with Shape medical polymer sponge embolization as adjunct therapy for rapid aortic remodeling in the multi-modal management of complex persistent large false lumens following aortic dissection
Source: J Vasc Surg Cases Innov Tech. 2025 Jul 7;11(5):101913. doi: 10.1016/j.jvscit.2025.101913 (PMC12361994; doi:10.1016/j.jvscit.2025.101913)
Supplement: Supplementary Table III — Detailed surgical history for the patient described in Case 3. [file mmc3.docx]

**Supplementary Table 3.**

| **CASE 3 Detailed Surgical History** | |
| --- | --- |
| **Month/Year** | **Indication/Procedure/Devices** |
| September 2024 | Patient presented with Acute TAAD with RLE Malperfusion. Underwent total arch replacement with a Terumo Thoraflex Ante-Flo, 32mm stent and a trifurcated graft with transposition of the L vertebral artery to the L SCA limb of the graft. Aortic valve resuspended. Right EIA and CFA dissection treated with 9mmx60mm self expanding Boston Scientific Stent. R SFA cannulation site for perfusion closed with 8mmx5cm Viabahn stent from the contralateral access after RLE perfusion was restored. |
| October 2024 | Patient re-presented with complaints of abdominal pain, early satiety, and post prandial pain with CTA evidence of dynamic complete collapse of true lumen in paravisceral segment. Patient taken back to OR to re-expand true lumen from Zone 3-5 with three GORE cTAG devices working downward from Thoraflex stent, 34mmx100mm, 28x150mm, and a junctional 37mmx100mm. |
| December 2024 | Patient again presented with a shortness of breath in the setting of a heart failure exacerbation (EF 15% from 25% on discharge after initial presentation) at which time CTA chest demonstrated large Type 1b endoleak and sac expansion by 3mm compared to scan from one month prior. Patient also had a left sided pleural effusion which was tapped by the medical team and serosanguinous fluid was drained. With concern for impending rupture and to prevent rapid sac expansion, patient was taken to OR for embolization of sac with multiple agents. Endoplugging began with a A 27mmx10mm GORE iliac limb which was placed in FL. The limb was accessed to place 30 Shape Memory IMPEDE-FX Embolization Plugs into the FL. The limb was then plugged with an 18mmx22mm Amplatzer Plug. |
| Abbreviations: TAAD-Type A Aortic Dissection. RLE-Right Lower Extremity. SCA-Subclavian Artery. EIA-External Iliac Artery. CFA-Common Femoral Artery. SFA-Superficial Femoral Artery. CTA-Computed Tomography Angiogram. cTAG- Conformable Thoracic Stent Graft. FL- False Lumen. Terumo-Terumo Medical Corporation. GORE-W.L. Gore & Associates, Flagstaff AZ. Shape Memory-Shape Memory Medical Inc., San Jose, CA. | |
